# Investigations on metabolic diseases of horses in Egypt

**DOI:** 10.3389/fvets.2025.1591090

**Published:** 2025-08-18

**Authors:** Amal M. Aboelmaaty, Ahmed M. Ahdy, Sabry El-khodery, Magdy Elgioushy

**Affiliations:** ^1^Animal Reproduction and AI Department, Veterinary Research Institute, National Research Centre, Cairo, Egypt; ^2^Department of Parasitology and Animal Diseases, Veterinary Research Institute, National Research Centre, Giza, Egypt; ^3^Department of Medicine and Infectious Medicine, Faculty of Veterinary Medicine, Mansoura University, Mansoura, Egypt; ^4^Department of Animal Medicine, Faculty of Veterinary Medicine, Aswan University, Aswan, Egypt

**Keywords:** Cushing’s disease, MARES, insulin, glucose, nitric oxide, antioxidants

## Abstract

**Introduction:**

Obesity is a significant risk factor that predisposes horses to laminitis, equine metabolic syndrome, arthritis, heart disease, and respiratory issues.

**Methods:**

Mares showing overweight or laminitis (*N* = 30), different BCS (*N* = 90) weighing 350–550 Kg were subjected to clinical and rump fat assessments. Blood samples were collected to measure circulating estradiol, progesterone, cortisol, insulin, insulin-like growth factor-1 (IGF-1), and leptin. Additionally, glucose, cholesterol, triglycerides, total proteins, albumin, nitric oxide (NO), lactate dehydrogenase (LDH), glutathione reduced, catalase, and serum amyloid A were measured. Statistical comparisons among mares were based on laminitis, hyperinsulinemia, overweight, the combination of hyperinsulinemia and overweight, and rump fat levels.

**Results:**

Laminitis was associated with lower glucose levels (4.83 ± 0.16 *vs.* 5.56 ± 0.52 mmol/L; *p =* 0.002) and body weight (380.0 ± 10.95 *vs.* 447.6 ± 65.98 kg; *p =* 0.02), as well as higher albumin (3.78 ± 0.01 *vs.* 3.11 ± 0.62 g/dL; *p =* 0.015), catalase (193.64 ± 69.29 *vs.* 37.45 ± 7.57 U/mL; *p =* 0.001), IGF-1 (29.35 ± 17.31 *vs.* 20.29 ± 6.48 ng/mL; *p =* 0.043), and the glucose/insulin ratio (0.77 ± 0.68 *vs.* 0.44 ± 0.030; *p >* 0.05). Obesity (>450 Kg) was associated with increased glucose levels (5.60 ± 0.54 *vs.* 5.15 ± 0.39 nmol/L; *p =* 0.018) and reduced IGF-1 (16.44 ± 4.51 *vs.* 28.24 ± 3.90 ng/mL; *p <* 0.001), albumin (2.98 ± 0.67 *vs.* 3.46 ± 0.53 g/dL; *p =* 0.041), albumin/globulin ratio (0.72 ± 0.07 *vs.* 0.98 ± 0.11; *p =* 0.048), and glucose/insulin ratio (0.34 ± 0.14 *vs.* 0.71 ± 0.58; *p =* 0.009). Hyperinsulinemia (>20 μU/L) was associated with significant reduction in albumin (2.67 ± 0.59 *vs.* 3.56 ± 0.37 g/dL; *p =* 0.0001), albumin/globulin ratio (0.57 ± 0.24 *vs.* 1.01 ± 0.32 g/dL; *p =* 0.0001), catalase (17.23 ± 2.24 *vs.* 100.67 ± 30.99 U/mL; *p =* 0.021), NO (19.23 ± 2.24 *vs*. 21.35 ± 1.29 mmoL/mL; *p* = 0.002), glucose (5.44 ± 0.53 *vs.* 5.61 ± 0.38 mmol/L; *p =* 0.038), and glucose/insulin ratio (0.18 ± 0.04 *vs.* 0.72 ± 0.41; *p =* 0.0001). In contrast, globulin levels were significantly elevated (5.02 ± 1.02 *vs*. 3.85 ± 1.10 g/dL; *p* = 0.0001). The combination of hyperinsulinemia and obesity was associated with significant decreases in (*p =* 0.0001) albumin, albumin/globulin, and IGF-1, and increases in LDH, NO, globulins (*p =* 0.006). Additionally, NO levels were significantly reduced in hyperinsulinemia mares with lower body weight (*p =* 0.0001).

**Conclusion:**

Obesity, whether assessed by rump fat or overweight, is not always associated with hyperinsulinemia or with metabolic or endocrinologic abnormalities in mares. Conversely, hyperinsulinemia is not always associated with obesity but is related to insulin resistance and dysregulation.

## Introduction

Equine obesity, overweight, elevated body condition score, and Cushing’s disease are associated with metabolic and endocrine disturbances (equine metabolic syndrome) such as hyperleptinemia, hyperinsulinemia, hyperlipidemia, and hypertriglyceridemia. These conditions are particularly common in performance horses that cease regular training, riding, or jumping without appropriate diet management—especially diets high in carbohydrates (1, 2). Adiposity in mares can be determined using body condition scoring (3), cresty neck scoring (4, 5), and ultrasonography to measure rump fat thickness (6–8). Morphometric measurements were used to estimate adiposity across different horse breeds (9, 10). Alterations in leptin (1, 6, 7) are the key features of the metabolic syndrome in mares. Leptin is one of the important adipocyte hormones that plays a central role in appetite regulation (11). Most hyperleptinemic obese horses are also hyperinsulinemic and developed insulin resistance (12). In Andalusian horses, hyperleptinemia has been associated with neck adiposity and a body condition score (BCS) greater than 6 (4, 5). In the normal or metabolic syndrome conditions, hyperinsulinemia results from either oversecreting of insulin or hepatic dysfunction in clearing excess overproduced insulin, which is responsible for insulin resistance secondary to changes in body weight (13).

Excess adiposity has been associated with alterations in circulating adipokines (14, 15). Leptin regulates appetite (11), while adiponectin manages blood glucose, improves insulin sensitivity and high-density lipoproteins, lowers triglycerides, regulates hepatic glucose production, and promotes glucose uptake in muscles (16). Acute phase proteins (APPs) are inflammatory markers that exhibit significant changes in serum concentration during inflammatory disorders associated with obesity (17). Durham et al. (18) had mentioned the ECEIM consensus statement on equine metabolic syndrome, the clinical and differential diagnosis, and the nutritional and exercise management (18).

Disturbances in proinflammatory cytokines, insulin, lipids, and fat deposition have been shown to predispose mares to laminitis and alter the reproductive function (19). The increase of insulin or hyperinsulinemia more than 20 mU/L was associated with laminitis and Cushing’s disease in ponies (20, 21) and horses (22). Hyperinsulinemia of horses was associated with high ferritin in the blood of horses (23). Hyperinsulinemia associated with pituitary pars intermedia dysfunction (PPID) and/or equine metabolic syndrome (EMS) is well-documented as a major risk factor for laminitis in horses (24, 25). Fewer than 10% of ponies with above-optimal BCS and elevated cresty neck scores with regionalized adiposity and chronic laminitis showed hyperinsulinemia, hypertriglyceridemia, and hyperglycemia (26). Obesity in mares, manifested by elevated BCS and insulin, had higher concentrations of inflammatory cytokines compared to obese stallions and geldings (27, 28). Laminitis, hyperinsulinemia, hyperleptinemia, hyperlipidemia, and oxidative stress dysregulations were among the risks and consequences of obesity in horses (26, 29).

This study hypothesized that back fat thickness and some adipokines could serve as markers of obesity in mares via investigating the association of hyperinsulinemia and overweight, laminitis, with circulating insulin, insulin-like growth factor-I (IGF-1), catalase, glutathione reduced (GSH), nitric oxide (NO), total cholesterol, glucose, total proteins, albumin, globulins, and lactate dehydrogenase (LDH). Additionally, the association of rump fat and cortisol, estradiol, progesterone, IGF-1, leptin, and serum amyloid A (SAA) was examined.

## Methodology

The study protocol was approved by the Animal Care and Use Committee of the Faculty of Veterinary Medicine, Mansoura University (MU-ACUC-VM.R.23-10.129).

### Animals

A total of 120 mares, aged between 5 and 12 years and weighing 350–550 kg, were included. **Experiment 1:** Arabian mares (*n* = 30), weighing between 350 and 550 kg, were selected from a private stud. They were housed individually in separate boxes and under the same management conditions, and they exhibited signs of hair overgrowth, laminitis, and overweight. Mares underwent thorough clinical examinations, including body weight estimation. Blood samples were collected from animals showing symptoms and controls. Cyclic, non-lactating Arabian brood mares exhibited no clinical symptoms other than those specified in the study.

All brood mares used for training and sports were non-pregnant and non-lactating.

### Blood sampling, hormone, and biochemical profiles

Blood samples were collected in sodium fluoride vacuum tubes for glucose measurement and in plain vacuum tubes for analysis of insulin, insulin-like growth factor-I, total proteins, albumin, globulin, total cholesterol, lactate dehydrogenase (LDH), catalase, reduced glutathione (GSH), and nitric oxide (NO).

Apparently healthy mares were classified into two groups: those presenting symptoms of laminitis (Cushing’s group) and those of overweight mares without laminitis (obese group). Mares were classified based on estimated body weight into two groups: obese (>450 kg BW) and control (<400 kg BW). Mares were based on insulin levels, with hyperinsulinemia defined as insulin levels >20.0 mU/L (30), and controls having <20 mU/L. Moreover, based on both body weight and insulin levels, mares were subdivided into four groups: hyperinsulinemic-light, hyperinsulinemic-obese, normoinsulinemic-light, and normoinsulinemic-obese.

### Experiment 2

Brood Arabian mares (*n* = 90) maintained at Horsey studs under the Ministry of Interior, Egypt, were included in the study. Body condition score (BCS) was assessed using a 9-point scale, ranging from 1 (very poor) to 9 [extremely fat; (3, 31)]. Back fat depth was measured (7, 32) using an ultrasound scanner (SonoVet R3 pulsed-wave Doppler ultrasound scanner, Madison, Samsung, South Korea) equipped with 12 MHz linear-array transducer. The site was scanned, and the same site was measured in all horses. Body weight was estimated in horses using the equation of Carroll and Huntington (33): BW (kg) = (Heart girth measurement in cm)^2^ × length measurement in cm/11,900. Considering the overweight to obese body condition score (BCS) of 7.8/9 ± 0.18, where BCS 1 indicated an emaciated condition and BCS 9 represents obesity (3, 31). Mares were categorized based on their BCS and back fat into three groups: obese (BCS > 7 and RF > 7 mm), optimal body condition (BCS > 3 to ≤ 7 and RF > 4 to ≤ 7 mm), and light (BCS ≤ 3 and RF ≤ 4 mm).

### Blood sampling, hormonal, and nitric oxide measurements

Blood samples were collected from all mares via the jugular vein using plain vacutainer tubes for hormone and biochemical assays, and sodium fluoride vacutainer tubes for glucose estimation. The collected serum and plasma samples were stored at −20°C until the hormone assays were performed. Estradiol (E2, EIA-2693), progesterone (P4, EIA-1561), and cortisol (EIA-1887) were assayed using competitive ELISA kits (DRG International, Inc., USA). The sensitivity of the assays for E2, P4, and cortisol was 9.714 pg/mL, 0.045 ng/mL, and 0.025 pg/mL, respectively. The intra- and inter-assay precisions were 2.71% and 6.72% for estradiol (E2). 6.86% and 5.59% for progesterone (P4), and 8.1% and 6.6% for cortisol, respectively. Commercial Sandwich ELISA kits were used to assay horse insulin (BYEK3072, Chongqing Bioseps Co., Ltd, China), horse insulin-like growth factor-I (IGF-1, BYEK3073; Chongqing Bioseps Co., Ltd, China), horse leptin (NOVA Bioneovan Co., Ltd, 18 Keyuan Road, DaXing Industry Zone, Beijing, China), and horse serum amyloid A (SAA, NOVA Bioneovan Co., Ltd. 18 Keyuan Road, DaXing Industry Zone, Beijing, China). The assay ranges were as follows: insulin, 0.8–30.0 mU/L; IGF-1, 0.1–20.0 ng/mL; leptin, 35–2000 pg/mL; and SAA, 33–2000 pg/mL. The minimum detection limits of the assays were: insulin, 0.06 mU/L; IGF-1, 0.5 ng/mL; leptin, 8 pg/mL; and SAA, 35 pg/mL. The assay percussions were <10 % and <12% for all horse-specific ELISA kits.Commercial colorimetric kits (Egyptian Co. for Biotechnology, Spectrum Diagnostics, Egypt) were used to measure total proteins (Cat. No. 310–002; sensitivity: 1.0 g/dL; intra- and inter-assay precisions: 2.31 and 3.33%, respectively), albumin (Cat. No. 210–002; sensitivity: 1.0 g/dL; precisions: 2.44 and 2.65%), glucose (Cat. No. 250–002; sensitivity: 0.27 nmol/L; precisions: 1.09 and 1.13%), cholesterol (Cat. No. 230–002; sensitivity: 5.0 mg/dL; precisions: 1.13 and 1.13%), and triglycerides (Cat. No. 314–002; sensitivity: 5.0 mg/dL; precisions: 1.31 and 1.41%) according to the method of Megerssa et al. (34).

Nitric oxide metabolites (NOMs), catalase, and glutathione reduced were analyzed in blood sera (BioDiagnostics, Egypt). Lactate dehydrogenase (LDH) was assayed using enzymatic kinetic-specified test commercial kits (Salucea, Italy).

### Statistical analysis

Data are presented as means ± standard deviation (SD). An independent sample t-test was used to compare differences between two groups, while one-way ANOVA was used for comparisons among more than two groups.

A one-way ANOVA was conducted to compare animals with Cushing’s disease and control groups based on insulin status (hyperinsulinemia or low insulin), different rump fat thicknesses, and hyperinsulinemia with obese or light, and their respective obese and light controls with normal insulin levels. Duncan’s Multiple Range Test was used to compare significant means at *p <* 0. 05. Pearson correlation coefficients were also calculated to assess the relationships between variables.

## Results

### Experiment 1

Results revealed that mares showing laminitis and hair overgrowth (Cushing’s; Table 1) had significantly lower glucose levels (4.83 ± 0.16 *vs.* 5.56 ± i0.52 mmol/L; *p =* 0.002) and body weight (380.0 ± 10.95 *vs.* 447.6 ± 65.98 kg; *p =* 0.02), but higher albumin (3.78 ± 0.01 *vs.* 3.11 ± 0.62 g/dL; *p =* 0.015), catalase (193.64 ± 69.29 *vs.* 37.45 ± 7.57 U/mL; *p =* 0.001), IGF-1 (29.35 ± 7.07 *vs.* 20.29 ± 6.48 ng/mL; *p =* 0.043), and glucose/insulin ratio (0.77 ± 0.68 *vs.* 0.44 ± 0.30; *p =* 0.013) than apparently healthy controls.

**Table 1:** Mean ± SD of different blood biochemicals and metabolites, hormones, and antioxidants in Arabian mares showing Cushing’s syndrome.

Parameter	Cushing’s		Control		*p*-value
*N*	6		24		
Parameters	Mean ± SD	Range	Mean ± SD	Range	
BW	380.0 ± 10.95	370–390	447.6 ± 65.98	305–500	0.002
Total proteins g/dl	7.62 ± 0.08	7.55–7.70	7.37 ± 0.87	6.20–8.66	0.493
Albumin g/dl	3.78 ± 0.01	3.78–3.79	3.11 ± 0.62	2.18–3.99	0.015
Globulin g/dl	3.84 ± 0.08	3.77–3.91	4.26 ± 1.185	2.50–5.83	0.396
Albumin/globulin	0.98 ± 0.02	0.97–1.00	0.83 ± 0.38	0.42–1.48	0.324
Cholesterol mg/dl	116.9 ± 11.08	106.79–127.01	117.2 ± 12.13	89.89–130.65	0.963
Cholesterol mmol/L	2.94 ± 0.27	2.68–3.19	2.94 ± 0.30	2.26–3.28	0.946
LDH U/L	8.09 ± 2.11	6.07–10.12	6.83 ± 3.06.13	2.02–10.12	0.182
Catalase U/mL	193.64 ± 69.29	14.45–372	37.45 ± 7.57	3.74–121.39	0.001
GSH mg/dL	54.3 ± 2.8	51.79–56.79	54.0 ± 4.6	45.06–61.93	0.856
NO mmol/L	19.59 ± 2.60	17.22–21.97	20.68 ± 2.02	16.73–22.87	0.274
IGF-1 ng/mL	29.35 ± 17.31	13.55–45.15	20.29 ± 6.48	115.04–36.23	0.043
Insulin mU/L	18.49 ± 16.34	3.57–33.42	18.05 ± 9.40	4.62–31.64	0.900
Glucose mmol/L	4.83 ± 0.16	4.68–4.98	5.56 ± 0.52	4.33–6.06	0.002
Glucose/Insulin ratio	0.77 ± 0.68	0.14–1.39	0.44 ± 0.30	0.14–1.14	0.013
Insulin/glucose ratio	3.93 ± 3.52	0.88–7.31	3.36 ± 2.04	0.72–7.31	0.457

Obese Arabian mares (BW > 450 Kg) had higher glucose levels (5.60 ± 0.54 *vs.* 5.15 ± 0.39 mmol/L; *p =* 0.018) and globulin (4.62 ± 1.17 *vs.* 3.96 ± 1.16 g/dL; *p =* 0.03), but lower IGF-1 (16.44 ± 4.51 *vs.* 28.24 ± 3.90 ng/mL; *p <* 0.001), catalase (CAT, 41.7 ± 37.8 *vs.* 100.4 ± 47.4 U/L; *p =* 0.041), albumin (2.98 ± 0.67 *vs* 3.46 ± 0.53 g/dL; *p =* 0.041), albumin/globulin ratio (0.72 ± 0.07 *vs.* 0.98 ± 0.11; *p =* 0.048), and glucose/insulin ratio (0.34 ± 0.14 *vs.* 0.71 ± 0.58; *p =* 0.009) than their controls (BW < 400 kg; Table 2).

**Table 2:** Mean ± SD of different blood biochemicals and metabolites, hormones, and antioxidants in Arabian obese and control mares.

Parameters	Obese (BW>400 kg)		Control (BW<400 kg)		*p*-value
*N*	21		9		
Parameters	Mean ± SD	Range	Mean ± SD	Range	
BW/kg	476.57 ± 24.04	450–500	363.75 ± 36.44	305–390	0.0001
Total proteins g/dl	7.59 ± 0.85	6.35–8.66	7.43 ± 0.79	6.2–8.26	0.423
Albumin g/dl	2.98 ± 0.67	2.18–3.99	3.46 ± 0.53	2.58–3.79	0.041
Globulin g/dl	4.62 ± 1.17	2.80–6.40	3.96 ± 1.16	2.50–5.68	0.032
Albumin/globulin	0.72 ± 0.07	0.35–1.27	0.98 ± 0.11	0.45–1.48	0.048
Cholesterol mg/dl	119.7 ± 17.63	89.89–151.50	121.1 ± 9.51	106.79–130.65	0.716
Cholesterol mmol/L	3.01 ± 0.44	2.26–3.81	3.04 ± 0.56	2.68–3.28	0.265
LDH U/mL	6.65 ± 3.23	2.02–10.12	7.08 ± 2.31	4.05–10.12	0.566
CAT U/L	41.70 ± 37.81	6.74–121.39	100.43 ± 47.43	6.74–372.83	0.026
GSH mg/dL	54.62 ± 5.33	45.06–61.93	54.56 ± 1.91	51.79–56.79	0.956
NO mmol/L	20.78 ± 1.33	18,399–22.77	19.69 ± 2.86	16.73–22.87	0.09
IGF-1 ng/ml	16.44 ± 4.50	7.00–21.60	28.24 ± 13.52	13.55–45.15	0.001
Insulin mU/L	19.17 ± 7.46	10.42–31.64	18.23 ± 14.78	3.57–33.42	0.725
glucose mmol/L	5.60 ± 0.54	4.33–6.06	5.15 ± 0.39	4.68–5.69	0.018
glucose/Insulin ratio	0.34 ± 0.14	0.14–0.57	0.71 ± 0.58	0.14–1.39	0.009
Insulin/glucose ratio	3.58 ± 0.83	1.77–7.31	3.56 ± 2.95	0.72–7.14	0.975

Hyperinsulinemic mares (insulin >20 mU/L; 29.16 ± 3.87 mU/L) exhibited lower levels of albumin (2.67 ± 0.59 *vs.* 3.71 ± 0.37 g/dL; *p =* 0.0001), albumin/globulin ratio (0.57 ± 0.24 *vs.* 1.12 ± 0.32 g/dL; *p =* 0.0001), CAT (44.7 ± 3.03 *vs.* 100.43 ± 32.79 U/mL; *p =* 0.021), NO (19.23 ± 2.24 *vs.* 21.35 ± 1.29 mmoL/mL; *p =* 0.002), glucose (5.23 ± 0.53. *vs.* 5.59 ± 0.38 mmol/L; *p =* 0.049), and glucose/insulin ratio (0.18 ± 0.04 *vs.* 0.72 ± 0.41; *p =* 0.0001), but significantly higher levels of globulin (5.02 ± 1.02 *vs.* 3.45 ± 1.10 g/dL; *p =* 0.0001) and insulin/glucose ratio (5.72 ± 1.36 *vs.* 1.78 ± 0.77; *p =* 0.041; Table 3).

**Table 3:** Mean ± SD of different blood biochemicals and metabolites, hormones, and antioxidants in Arabian mares with hyperinsulinemia (>20 mU/L) and control (<20 mU/L).

Insulin l	Hyperinsulinemia >20 mU/L		Control <20 mU/L		*p*-value
*N*	12		18		
Parameters	Mean ± SD	Range	Mean ± SD	Range	
BW	426.20 ± 74.05		443.33 ± 50.99		0.264
Total proteins g/dl	7.68 ± 0.73	6.63–8.64	7.40 ± 0.89	6.2–7.92	0.159
Albumin g/dl	2.67 ± 0.59	2.18–3.78	3.71 ± 0.37	3.49–3.992	0.0001
Globulin g/dl	5.02 ± 1.02	3.77–6.4	3.45 ± 1.10	2.50–4.10	0.004
Albumin/globulin	0.57 ± 0.24	0.35–1.00	1.12 ± 0.32	0.85–1.48	0.0001
Cholesterol mg/dl	117.98 ± 21.94	89.9–151.5	123.31 ± 4.51	117.5–127.0	0.265
Cholesterol mmol/L	2.97 ± 0.54	2.26–3.81	3.06 ± 0.11	2.92–3.19	0.698
LDH U/mL	6.65 ± 3.23	2.02–10.12	7.08 ± 2.316	4.05–10.12	0.404
CAT U/L	44.70 ± 7.35	6.74–121.39	100.43 ± 32.79	6.74–372.83	0.021
GSH mg/dL	53.25 ± 4.98	45.06–59.66	55.73 ± 3.51	50.93–61.93	0.191
NO mmol/L	19.23 ± 2.24	16.73–22.77	21.35 ± 1.29	20.78–22.87	0.002
IGF-1 ng/ml	18.99 ± 10.18	7.0–36.23	23.18 ± 10.70	15.04–45.15	0.215
Insulin mU/L	29.16 ± 3.87	23.77–33.42	9.29 ± 4.72	3.57–14.86	0.0001
Glucose mmol/L	5.23 ± 0.53	4.33–5.74	5.59 ± 0.38	4.89–6.06	0.038
Glucose/Insulin ratio	0.18 ± 0.04	0.14–0.24	0.72 ± 0.41	0.39–1.39	0.0001
Insulin/ glucose ratio	5.72 ± 1.36	4.14–7.31	1.78 ± 0.77	0.72–2.59	0.0001

Hyperinsulinemia-obese mares and hyperinsulinemia-light (low body weight (BW)) mares had significantly lower albumin (*p =* 0.0001) and albumin//globulin (*p =* 0.0001), but higher globulin levels (*p =* 0.006) compared to their respective control groups. CAT activity (*p =* 0.004) and NO (*p =* 0.0001) levels were maximum in mares with normal insulin-light BW and were minimum in hyperinsulinemia-light mares. IGF-1 declined (*p =* 0.005) in obese mares with hyperinsulinemia and normal insulin (Table 4). Obese mares with normal insulin had the highest glucose concentrations (*p =* 0.004). In light Arabian mares with normal insulin (Table 4), the lowest (*p =* 0.001) insulin concentrations were associated with the highest (*p =* 0.0001) glucose/insulin ratio.

**Table 4:** Blood biochemicals and metabolites, hormones, and antioxidants in Arabian mares with hyperinsulinemia (insulin >20 mIU), overweight (BW > 400 kg), and control (BW < 400 kg), and normal insulin (insulin <20 mIU).

Parameter	Hyperinsulinemia Mean ± SD (Range)		Normal Insulin Mean ± SD (Range)		*p*-value
	Obese	Light	Obese	Light	
*N*	6	6	12	6	
Body weight /Kg	479 ± 26^c^ (450–500)	348 ± 60^a^ (305–390)	475 ± 26^c^ (450–500)	380 ± 14^b^ (370–390)	0.001
Total proteins g/dl	7.54 ± 1.02^ab^ (6.63–8.64)	7.90 ± 0.50^b^ (7.55–8.26)	7.63 ± 0.96^ab^ (6.35–8.66)	6.95 ± 1.06^a^ (6.2–7.7)	0.225
Albumin g/dl	2.33 ± 0.21^a^ (2.18–2.57)	3.18 ± 0.84^b^ (2.58–3.78)	3.47 ± 0.48^bc^ (2.83–3.99)	3.74 ± 0.06^c^ (3.70–3.79)	0.001
Globulin g/dl	5.22 ± 1.17^b^ (4.06–6.40)	4.73 ± 1.35^b^ (3.77–5.68)	4.17 ± 1.25^ab^ (2.80–5.83)	3.21 ± 0.99^a^ (2.50–3.91)	0.006
Albumin/globulin	0.47 ± 0.15^a^ (0.35–0.63)	0.73 ± 0.39^ab^ (0.45–1.0)	0.91 ± 0.33^b^ (0.49–1.27)	1.22 ± 0.36^c^ (0.97–1.48)	0.001
Cholesterol mg/dl	117.5 ± 31.3 (89.89–151.5)	118.7 ± 16.87 (107–131)	121.1 ± 5.33 (116–126)	123.5 ± 4.92 (120–127)	0.879
Cholesterol mmol/L	2.95 ± 0.66 (2.26–3.81)	2.98 ± 0.31 (2.68–3.28)	3.05 ± 0.12 (2.92–3.18)	3.11 ± 0.09 (3.02–3,019)	0.698
LDH U/L	7.07 ± 1.06 (4.05–10.12)	5.36 ± 1.44 (6.07–8.10)	7.09 ± 3.43 (2.02–10.12)	7.08 ± 3.17 (4.05–10.12)	0.153
Catalase U/L	22.8 ± 14.72^a^ (6.74–35.65)	10.59 ± 4.02^a^ (6.74–14.45)	55.87 ± 76.33^a^ (11.56–121.39)	190.3 ± 55.04^b^ (7.71–372.8)	0.004
GSH mg/dL	53.02 ± 7.39 (45.06–59.66)	53.59 ± 2.54 (51.79–55.39)	55.83 ± 4.70 (50.93–61.93)	55.53 ± 1.79 (54.26–56.79)	0.452
NO mmol/L	20.74 ± 1.76^b^ (20.74–22.77)	16.97 ± 0.34^a^ (16.73–17.22)	20.81 ± 1.37^b^ (18.99–22.27)	22.42 ± 0.64^c^ (21.97–22.87)	0.0001
IGF-1 ng/ml	15.06 ± 6.42^a^ (7.0–21.6)	24.89 ± 12.42^bc^ (13.55–36.23)	17.47 ± 2.05^ab^ (15.04–20.52)	31.59 ± 14.85^c^ (18.03–45.15)	0.005
Insulin mU/L	27.03 ± 4.10^c^ (23.77–31.64)	32.36 ± 1.51^d^ (31.29–33.42)	13.28 ± 2.10^b^ (10.42–14.86)	4.09 ± 0.74^a^ (3.57–4.62)	0.0001
Glucose mmol/L	5.26 ± 0.69^a^ (4.33–5.74)	5.18 ± 0.55^a^ (4.68–5.69)	5.86 ± 0.14^b^ (5.74–6.06)	5.13 ± 0.16^a^ (4.98–5.27)	0.004
Glucose/Insulin	0.20 ± 0.05^a^ (0.14–0.24)	0.16 ± 0.02^a^ (0.14–0.18)	0.45 ± 0.08^b^ (0.39–0.57)	1.27 ± 0.14^c^ (1.14–1.39)	0.0001
Insulin/Glucose	5.31 ± 1.150^c^ (4.14–7.31)	6.32 ± 0.89^d^ (5.5–7.14)	2.27 ± 0.36^b^ (1.77–2.59)	0.79 ± 0.09^a^ (0.72–0.88)	0.0001

Superscript letters (a, b, c, d) within the rows indicate significance at *p* < 0.05.

**Figure 1:**
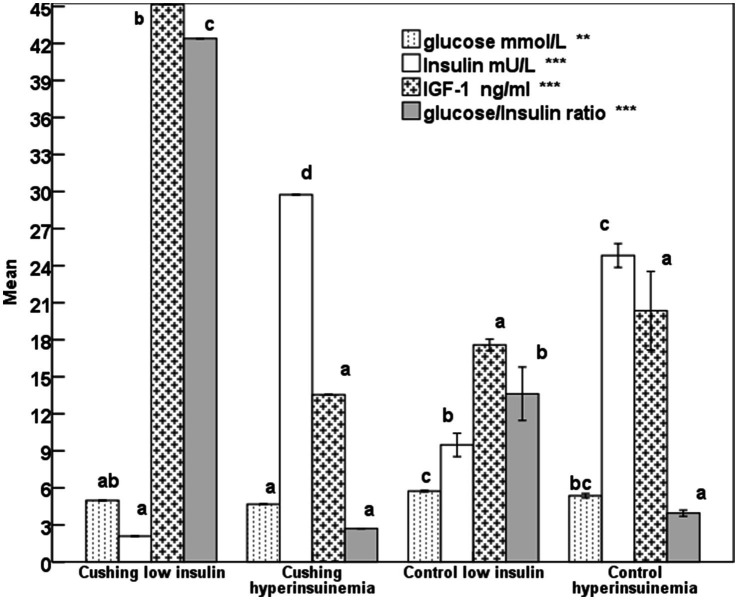
Mean glucose, insulin, insulin-like growth factor-1 (IGF-1), and glucose-insulin ratio in mares expressing Cushing’s syndrome and hyperinsulinemia with error bars. Superscript letters (a, b, c, d) indicate significance at *p* < 0.05, ** means *p*-value <0.001; *** means *p* < 0.0001.

The differences in glucose in mares presented with Cushing’s syndrome with hyperinsulinemia or low insulin are not significant (Figure 1), but were lower (*p* < 0.001) than the controls showing low or hyperinsulinemia. Insulin concentrations exceeded 24 mU/L (*p* < 0.0001) in hyperinsulinemia control mares or those with Cushing’s syndrome (Figure 1). Mares presenting Cushing’s syndrome obtained the highest (*p* < 0.0001) IGF-1 and glucose insulin ratio (Figure 1).

**Figure 2:**
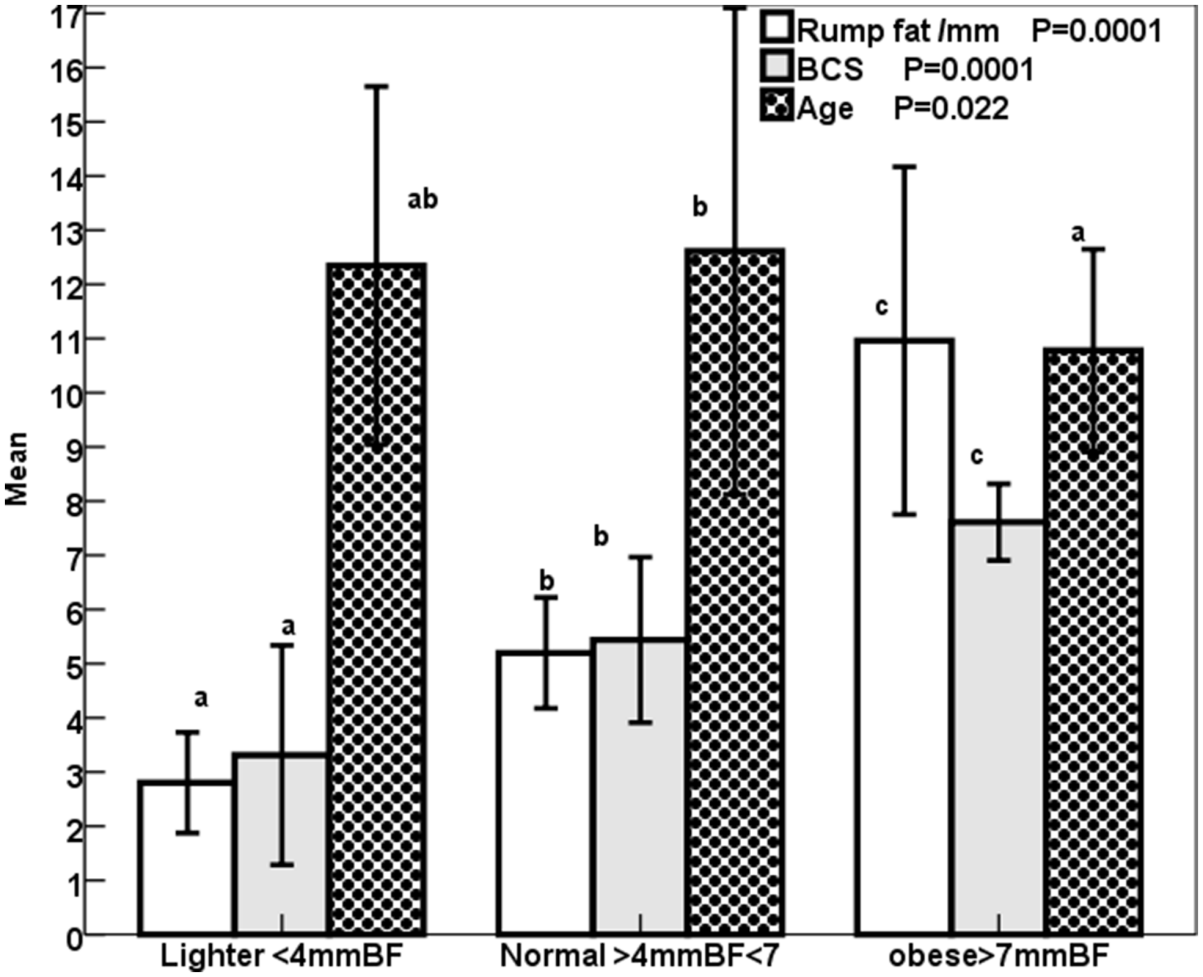
Mean ± SD BCS (1–9), rump fat/mm, and age /year of lighter, normal, and obese mares. Superscript letters (a, b, c) indicate significance at *p* < 0.05.

**Figure 3:**
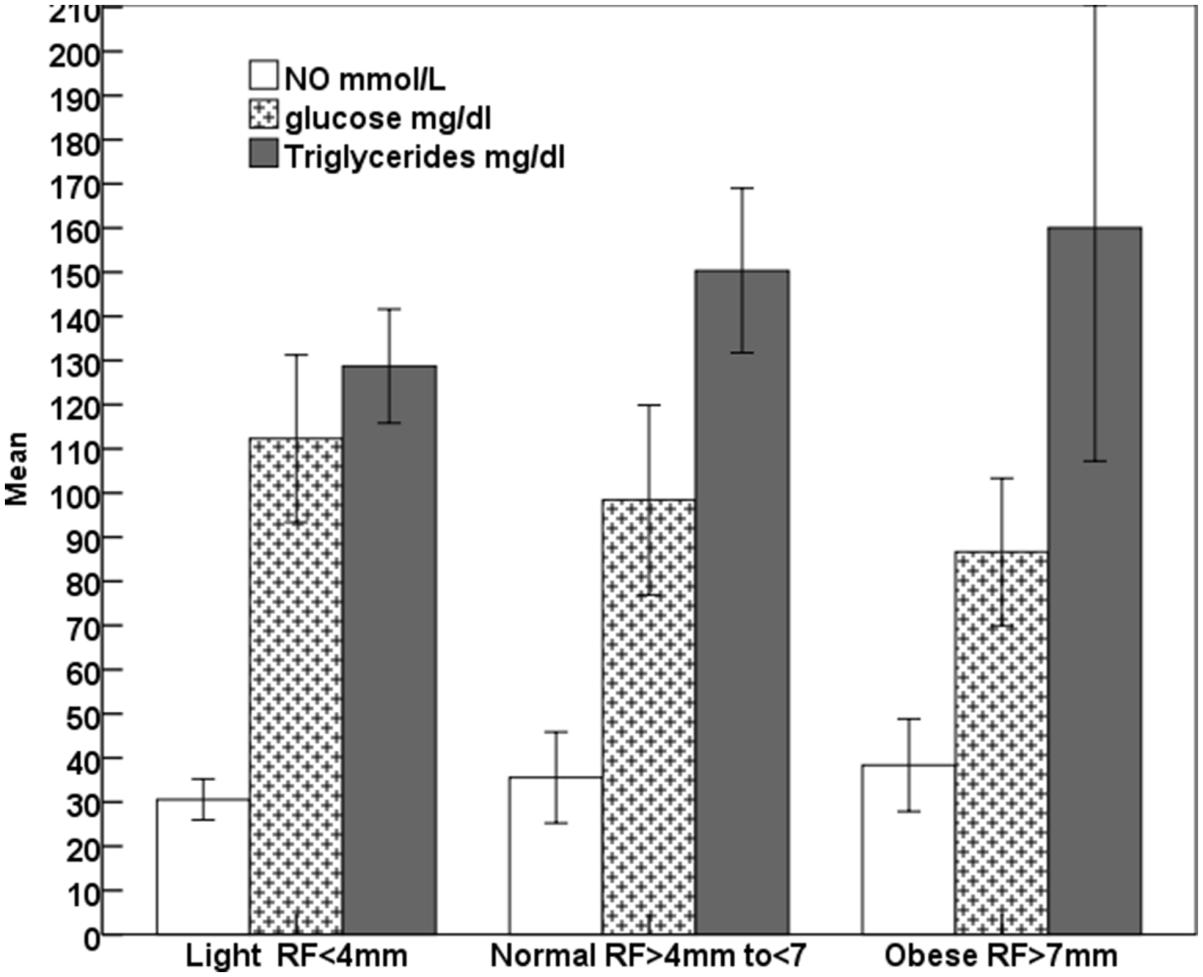
Mean ± SD glucose, triglycerides, and nitric oxide (NO) of lighter, normal, and obese mares.

The increase (*p* < 0.001) in the rump fat thickness (RF/mm) and BCS were parallel to each other from lighter to obese mares and also were associated with a significant decrease in age (*p* < 0.05; Figure 2). The increase in the rump fat from light to obese mares associated non-significant decrease in glucose levels and non-significant increases in triglycerides and nitric oxide (NO) levels (Figure 3). In addition to IGF-1, steroid hormones (Table 5) including cortisol, estradiol (E2), and progesterone (P4) did not vary in mares with increasing their rump fat and BCS, but E2 and P4 declined (*p* < 0.05) in obese mares. Obese mares had the lowest (Table 5) leptin (*p* < 0.05) and serum amyloid A (SAA) concentrations, and the highest nitric oxide (NO) concentrations (*p* < 0.01).

**Table 5:** Mean ± SD of different blood biochemicals and metabolites, hormones, and antioxidants in mares with different rump fat.

Parameter	Light RF<4 mm	Control RF>4- < 7	Obese RF>7	*p*-value
*N* samples	22	54	14	
Cortisol pg/ml	154.89 ± 11.38	152.10 ± 9.24	132.53 ± 14.71	0.610
E2 pg/ml	230.37 ± 37.30^b^	217.94 ± 21.19^ab^	136.73 ± 34.53^a^	0.115
P4 ng/ml	16.13 ± 3.56^b^	18.16 ± 2.74^ab^	15.16 ± 5.33^a^	0.313
IGF-1 ng/ml	23.36 ± 47.39	24.46 ± 26.88	17.87 ± 55.49	0.352
Leptin pg/ml	182.84 ± 28.69^ab^	228.16 ± 61.46^b^	100.77 ± 8.97^a^	0.002
SAA pg/ml	4,548 ± 259^a^	6,971 ± 709^b^	4,255 ± 238^a^	0.032

Superscript letters (a, b) within the rows indicate significance at *p* < 0.05.

Progesterone (*r* = − 0.23; *p* > 0.05), glucose (*r* = − 0.30; *p* < 0.01), and leptin (*r* = − 0.22) showed negative correlations with rump fat. SAA (*r* = 0.38; *p* < 0.01) and leptin (*r* = −0.30; *p* < 0.05) have positive correlations with BCS.

## Discussion

The body weight of Arabian horses is lower than that of non-Arabian breeds. Typically ranging from 350 to 400 kg. High subcutaneous rump fat and elevated body condition scores (BCS) were used as indicators for diagnosing obesity in horses (8). Resting sport horses recovering from musculoskeletal disease or those receiving increased carbohydrates in their diet develop adiposity in equine. Similar to our Arabian brood mares, as well as native and other breeds of brood and sport horses, the prevalence of obesity in pleasure Arabian and Quarter horses was lower than that of observed in ponies (35). In our results, Arabian mares with laminitis showed lower BW, glucose, and a slight increase in insulin and tended to have a high glucose/insulin ratio. In contrast, laminitis is one of the common risk factors of overweight or obesity (2). The mares that exhibited symptoms of laminitis and over hair growth in our study were lighter in weight but had insulin levels comparable to those without symptoms who were obese. Similarly, ponies showing laminitis had similar body weight and condition, glucose, and slightly lower insulin than those that showed no laminitis (36). Mares with equine metabolic syndrome had lower average body weight and a slight increase in BCS compared to their controls (37). This indicates that not all obese mares show metabolic syndrome. Although younger mares were found to have increased rump fat and higher BCS than control and thin mares of this study, neither age nor breed affected insulin, leptin, or serum amyloid A levels in horses fed a high-fat, hyperglycemic diet for 20 weeks—despite increased BCS, body fat mass, and cresty neck score (38). In agreement with our results, obese mares and those with a body condition score (BCS) greater than 7 had both normal insulin levels and hyperinsulinemia (29). In contrast to the presence of obese mares with normal insulin levels and others with hyperinsulinemia, insulin sensitivity was found to decrease with increasing BCS and body fat percentage in horses (28). Contrary to our results, over-conditioned mares and those with high BCS were hyperleptinemic (39). Though BCS and rump fat decreased in older horses of this study, horses aged >17 to 20 years had higher insulin levels than younger horses (29).

Surprisingly, laminitis reported in this study was not linked to obesity but associated with insulin levels <20 mU/L, lower body weight, and glucose levels, along with higher concentrations of albumin, catalase, glucose, insulin, and IGF-1 compared to obese mares. In ponies, laminitis was a risk factor for both hyperinsulinemia and hyperleptinemia (20).

The high glucose concentration in obese Arabian mares of this study was not associated with normal insulin levels, and hyperinsulinemia was also observed in obese horses without exceeding the normal glucose range (40). Contrary to our study, the increase of SAA and the positive correlation between SAA and BCS were reported in a similar study, where SAA increased in mares, stallions, and geldings with BCS > 7 and was minimum in those with BCS ranging from 5 to 6 whom also reported a correlation between SAA and BCS (27).

In obese ponies, the decrease in BCS from 8 to 5 was associated with a linear decrease in leptin over 14 weeks (41). Contrary to the low leptin concentrations in our obese and light mares compared to control, leptin concentrations increase with the increase in the amount of body fat mass (42) due to the presence of obesity-related leptin resistance (11).

The insignificant increase of globulin in obese mares compared to those having laminitis and the significant increase of globulins and the decrease of albumins indicate negative acute phase response in hyperinsulinemic mares. The increased globulins in the hyperinsulinemia obese mares of this study agreed with the increase in the steroid hormones binding globulins, inflammation, and insulin resistance in obese human patients (43) with high body mass index and excessive liver fat (44, 45). Steroid hormone-binding globulins improved lipid metabolism, restored insulin signal transduction, and reduced inflammation by lowering IL-6 during equine metabolic syndrome (46).

The insignificant decline of E2, P4, and cortisol concentrations in obese mares having high rump fat and BCS could be referred to the hyperinsulinemia and their binding with steroid binding globulin (45). The increased total cholesterol, leptin, and insulin with the normality of triglycerides in obese 18-day pregnant mares having nearly twice the body fat percent of their controls (47) disagree with the current results where obesity alone did not contribute to laminitis, hyperleptinemia, hyperinsulinemia, or increased total cholesterol, but hyperinsulinemia and hyperleptinemia may have contributed to the metabolic disorders in underconditioned or optimal-conditioned normal mares (7). However, the lower sample size within each group of mares showing Cushing’s (*N* = 6) compared to the control (*N* = 24) is considered acceptable for these symptoms in equines subjected to the same managerial conditions.

## Conclusion

Laminitis is not linked to obesity, increased body condition, hyperinsulinemia, or hyperglycemia. Instead, it is associated with a higher glucose/insulin ratio, catalase activity, and IGF1. Obesity correlates with decreased levels of albumin, albumin/globulin ratio, IGF-1, and glucose/insulin ratio. Hyperinsulinemia is linked to lower albumin, albumin/globulin ratio, catalase activity, glucose, and glucose/insulin ratio. Obese mares exhibited hyperinsulinemia along with reduced levels of albumin, albumin/globulin ratio, IGF-1, and glucose/insulin ratio. Over-conditioned mares exhibited similar levels of cortisol, estradiol, and progesterone, but had lower concentrations of leptin and IGF-1 compared to light mares and those in moderate optimal condition. Hyperinsulinemia and hyperleptinemia are not caused by obesity. IGF-1 can serve as a potential biomarker for Cushing’s disease, and hyperinsulinemia is not related to obesity.

## Data Availability

The raw data supporting the conclusions of this article will be made available by the authors, without undue reservation.
